# A Rare, Potentially Fatal Complication of a Common Virus: Epstein-Barr Virus-Induced Hemophagocytic Lymphohistiocytosis in Adolescence

**DOI:** 10.7759/cureus.94918

**Published:** 2025-10-19

**Authors:** Bakr Alhayek, Xiaowei Malone, Joelian Andrew Mislay, Ryan T Cardew, Asha Ramsakal

**Affiliations:** 1 Department of Internal Medicine, AdventHealth Tampa, Tampa, USA; 2 Department of Infectious Diseases, AdventHealth Tampa, Tampa, USA

**Keywords:** bone marrow biopsy, epstein-barr virus, hemophagocytic lymphohistiocytosis, pancytopenia, peritonsillar abscess, rare presentation

## Abstract

Hemophagocytic lymphohistiocytosis (HLH) is a rare, life-threatening hyperinflammatory syndrome that requires rapid recognition and treatment. We describe a 19-year-old female patient with a history of Graves’ disease who presented with Epstein-Barr virus (EBV) infection, severe pancytopenia, and was subsequently diagnosed with EBV-induced HLH. The presence of Graves’ disease, an autoimmune thyroid disorder, likely contributed to underlying immune dysregulation, predisposing the patient to an exaggerated immune response when exposed to EBV. This case highlights the importance of maintaining a high index of suspicion for HLH in patients with autoimmune conditions complicated by infection. Equally important is identifying the underlying trigger, as treatment strategies may differ, and include targeted therapies such as monoclonal antibodies in addition to, or in place of, traditional bone marrow-suppressive regimens.

## Introduction

Hemophagocytic lymphohistiocytosis (HLH) is a rare but life-threatening syndrome of uncontrolled immune activation that can lead to tissue damage and multi-organ failure [1]. It arises from impaired cytotoxic T and natural killer (NK) cell function, resulting in persistent macrophage activation, excessive cytokine release, and hemophagocytosis in tissues. Because no single feature is diagnostic, clinicians rely on frameworks such as the revised HLH-2004 criteria, which require five of the seven findings - fever (≥ 38.5 °C), splenomegaly (at least 2 cm below costal margin), cytopenias affecting at least two cell lineages, hypertriglyceridemia (≥ 3 mmol/L) or hypofibrinogenemia (≤ 1.5 g/L), hemophagocytosis in bone marrow or other tissues, hyperferritinemia (≥ 500 µg/L), or elevated soluble CD25 (≥ 2,400 U/ml) [2]. Despite these guidelines, diagnosis is often delayed due to nonspecific overlap with sepsis, malignancy, and autoimmune disease. Prompt recognition is critical, as untreated HLH carries a high risk of rapid deterioration [3].

Epidemiologic studies show mixed data on HLH incidence in different age groups. English and German cohorts report the highest rates of HLH in infancy, a trough in young adulthood, and a secondary rise in older age [4,5]. In contrast, United States data focusing only on adult HLH suggest a bimodal distribution with peaks in both young adults (16-30 years) and older adults (56-70 years) [6]. These differences highlight that while infancy carries the greatest risk, HLH can occur across the lifespan, with age propensities that may vary based on the population studied.

Epstein-Barr virus (EBV) infection is nearly universal, with global seroprevalence exceeding 95% [7]. While most cases manifest as self-limited infectious mononucleosis, progression to HLH is exceedingly rare, and the true incidence remains unknown [7]. Autoimmune diseases, including Graves’ disease, have also been reported as independent triggers for secondary HLH (sHLH), reflecting the role of immune dysregulation in disease pathogenesis [8].

We report the case of a 19-year-old female patient with a history of Graves’ disease who developed EBV-induced HLH, an uncommon presentation in an otherwise healthy young adult. This case highlights the diagnostic and therapeutic challenges of HLH, while underscoring the importance of recognizing coexisting autoimmune disease as a potential predisposing factor.

This article was previously posted to the Authorea preprint server on August 8, 2025 [9].

## Case presentation

**Figure 1:**
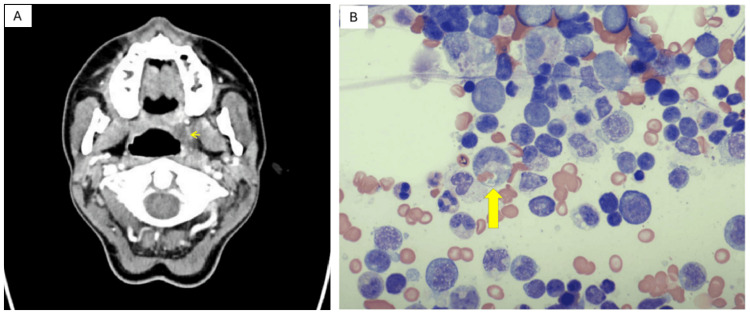
(A) Contrast-enhanced CT scan of the neck demonstrates findings consistent with acute tonsillitis and a dominant left peritonsillar abscess (yellow arrow) measuring up to 1.7 cm in diameter. (B) Bone marrow aspirate smear demonstrates hemophagocytosis, a hallmark of hemophagocytic lymphohistiocytosis (HLH). The image shows activated histiocytes with abundant cytoplasm engulfing hematopoietic elements (yellow arrow).

A 19-year-old White woman with a two-year history of Graves' disease treated with methimazole 10 mg orally twice daily reported six weeks of waxing-waning sore throat, fever, and malaise. Approximately five weeks before the index admission (hospital day -35), she received outpatient amoxicillin for presumed streptococcal pharyngitis. Five days later (day -30), a contrast CT of the neck showed a 1.7‑cm left peritonsillar abscess with bilateral tonsillitis and cervical lymphadenopathy (Figure 1A). She completed seven days of IV clindamycin 900 mg q8h plus dexamethasone 6 mg q12h, followed by a 10‑day oral course of clindamycin 300 mg four times daily and a methylprednisolone taper. She was well at discharge (day -5) without fever but returned five days later (day 0; admission day) with relapsed fever up to 39.4 °C/103 °F, severe odynophagia, palpitations, and fatigue. 

On arrival, she was tachycardic (heart rate: 135 beats/min), normotensive, and breathing comfortably. Examination showed 1 + symmetric erythematous tonsils without uvular deviation or trismus and mild splenomegaly; the remainder of the exam was unremarkable. Her laboratory workup on admission is shown in Table 1 (day 0). 

**Table 1:** Key laboratory values on day 0 and day 5 WBC, white blood cell count; ANC, absolute neutrophil count; AST, aspartate aminotransferase; ALT, alanine aminotransferase

Test (units)	Reference range	Day 0	Day 5
WBC (× 10³/µL)	4.0 - 11.0	1.73	1.15
ANC (× 10³/µL)	0.90 - 4.5	0.37	0.18
Hemoglobin (g/dL)	12.0 - 16.0	9.8	9.6
Platelets (× 10³/µL)	150 - 400	107	63
Sodium (mmol/L)	135 - 145	128	131
Creatinine (mg/dL)	0.60 - 1.20	0.40	0.30
AST (U/L)	5 - 40	316	322
ALT (U/L)	5 - 40	365	320
Alkaline phosphatase (U/L)	44 - 121	290	374
Total bilirubin (mg/dL)	0.2 - 1.0	1.0	3.40

Repeat neck CT scan demonstrated near-resolution of the abscess, sub-centimeter bilateral cervical lymphadenopathy, and diffuse thyroid enlargement. Empiric IV vancomycin 1.5 g q12h and cefepime 2 g q8h were started; methimazole was discontinued because of cytopenias. Broad infectious and autoimmune work‑up, including HIV, hepatitis A/B/C, herpes simplex virus (HSV), varicella-zoster virus (VZV), rapid plasma reagin (RPR), *Bartonella*, *Histoplasma*, *Blastomyces*, *Cryptococcus*, antinuclear antibody (ANA), and antineutrophil cytoplasmic antibody (ANCA), was negative.

Over the next six days, her fever, cytopenias, and liver‑enzyme elevations worsened despite antibiotics (Table 1, day 5). EBV polymerase chain reaction (PCR) measured 19,700 copies/mL. Ferritin peaked at 36,266 ng/mL and soluble IL‑2 receptor (sCD25) at 9,117 pg/mL (reference value: 175 to 858 pg/mL). Flow cytometry of peripheral blood identified an aberrant CD8⁺ T‑cell subset lacking CD5 (6.6% of events) with polyclonal T-cell receptor (TCR) β/γ rearrangements. In the context of acute EBV-associated hyperinflammation, loss of CD5 on a minority CD8⁺ subset is compatible with reactive cytotoxic T-cell activation and is not, by itself, diagnostic of a T-cell neoplasm; interpretation should rest on the total clinicopathologic picture.

Bone‑marrow biopsy revealed 95% cellularity with numerous hemophagocytic histiocytes (Figure 1B), decreased granulopoiesis, left‑shifted erythropoiesis, < 1% blasts by CD34/CD117, and positive EBV‑encoded RNA in‑situ hybridization. Reticulin stain was myelofibrosis grade 1 (MF‑1), and cytogenetics were normal.

Together, this patient met the revised HLH-2004 criteria for EBV-associated HLH with fever, cytopenias affecting at least two lineages, hemophagocytosis in bone marrow, hyperferritinemia, and elevated sCD25. Differential diagnoses included severe EBV mononucleosis, complicated bacterial infection, adult-onset Still's disease/macrophage activation syndrome (AOSD/MAS), acute leukemia/aplastic crisis, thrombotic thrombocytopenic purpura/disseminated intravascular coagulation (TTP/DIC), and autoimmune cytopenia; however, her hyperferritinemia, elevated sCD25, and marrow hemophagocytosis favored EBV-triggered HLH in this patient. 

High‑dose dexamethasone 8 mg IV q6h, intravenous immunoglobulin (IVIG) 1 g/kg daily × 2, and rituximab 640 mg weekly × 4 were initiated on day 6. Because the absolute neutrophil count (ANC) nadir reached 0.22 × 10³/µL, prophylactic acyclovir, fluconazole, and trimethoprim-sulfamethoxazole (TMP‑SMX) were given. The etoposide‑based HLH‑94 protocol [10] was deferred owing to profound pancytopenia. On day 25, emapalumab 1 mg/kg IV twice weekly was started, producing a transient improvement (peak ANC: 0.93 × 10³/µL; platelets: 128 × 10³/µL) before drug shortages limited escalation. She was transferred on day 32 to a regional HLH center; an eight‑week telephone follow‑up confirmed resolution of cytopenias. 

## Discussion

HLH can be classified into primary HLH (pHLH) and sHLH [11]. pHLH is caused by genetic mutations associated with immune dysfunction, such as LYST, SH2D1A, and PRF1, while sHLH is triggered by significant immune system insults, such as infections (e.g., EBV), malignancies, or autoimmune diseases ​[11]. The main clinical manifestations of HLH include fever, hepatosplenomegaly, lymphadenopathy, cytopenias, hyperferritinemia, hypertriglyceridemia, and/or hypofibrinogenemia ​[12]. In our case, the patient’s markedly elevated ferritin level served as the first clue leading to suspicion of HLH. It has been suggested that extremely high ferritin levels (e.g., > 10,000 µg/L) are more suggestive of HLH than other inflammatory conditions [2]. 

HLH can rise as a complication of EBV infection, and recent advancements have clarified the pathophysiological mechanisms underlying EBV-induced HLH. EBV-infected B cells activate cytotoxic T lymphocytes, resulting in hypercytokinemia and subsequent histiocytic cell activation ​[7]. Furthermore, EBV induces excessive activation of T and NK cells, driving the dysregulated production of cytokines, including IL-2, IFN-α, and IL-6, which play a central role in the hyperinflammatory state. An additional mechanism involves the EBV-induced expression of latent membrane protein 1 (LMP-1), which promotes IFN-α secretion and macrophage activation, potentially leading to immune dysregulation and HLH development [13]. Chronic EBV stimulation has also been implicated in the development of persistent HLH [13,14]. 

Once HLH has been diagnosed, therapy should be started as soon as possible. Of note, however, therapy should not be started until other hematological malignancies have been ruled out ​[15]. In North America and most of Europe, the treatment of HLH generally includes dexamethasone and etoposide based on the HLH-94 and HLH-2004 protocols ​[1,2,16,17]. However, in our case, this regimen was deferred due to profound pancytopenia due to the highly myelosuppressive effect of etoposide. Emapalumab, an IFN-γ-targeted monoclonal antibody, has been approved for pHLH in patients with refractory, recurrent, or progressive disease, or those intolerant to conventional therapy - including intolerance due to severe pancytopenia. Therefore, emapalumab was selected as the treatment of choice in this case [16]. Rituximab was also added, which has been shown to deplete EBV-harboring B cells and improve HLH ​[17]. Antifungal prophylaxis, anti-*Pneumocystis jirovecii *prophylaxis, antiviral prophylaxis, and IVIG replacement should all be considered per the above protocols ​[18]​ in patients with EBV-HLH. Of note, antiviral therapy targets the lytic (rather than latent) phase of EBV infection, which has not been shown to be significantly contributory to the EBV-related lymphoproliferative disorders and thus does not currently play a major role in most treatment protocols ​[19].

## Conclusions

This case underscores the importance of maintaining a high index of suspicion for HLH in patients with EBV infection who develop unexplained cytopenias, hyperferritinemia, or persistent fever. Although HLH most commonly affects infants and young children, it can present in adolescents and young adults, making timely recognition in this population critical. Our patient’s course highlights the diagnostic challenges of EBV-HLH, including its ability to mimic other infectious and hematologic conditions, and demonstrates the need for early bone marrow evaluation and prompt initiation of therapy. While traditional HLH treatment protocols rely on etoposide and corticosteroids, the profound pancytopenia in this case required an individualized approach that incorporated rituximab and, ultimately, emapalumab as steroid-sparing and targeted agents. This experience suggests that novel immunomodulatory therapies may represent valuable options in select patients with refractory EBV-associated HLH, although further study is needed to define their role. Ultimately, heightened awareness, early diagnosis, and patient-tailored strategies remain central to improving outcomes in this rare but life-threatening condition.
